# Genome-Wide Analysis of WRKY and NAC Transcription Factors in *Carica papaya* L. and Their Possible Role in the Loss of Drought Tolerance by Recent Cultivars through the Domestication of Their Wild Ancestors

**DOI:** 10.3390/plants12152775

**Published:** 2023-07-26

**Authors:** Erick Arroyo-Álvarez, Arianna Chan-León, Amaranta Girón-Ramírez, Gabriela Fuentes, Humberto Estrella-Maldonado, Jorge M. Santamaría

**Affiliations:** 1 Centro de Investigación Científica de Yucatán A.C., Calle 43 No. 130, Colonia Chuburná de Hidalgo, Mérida 97205, Yucatán, Mexico; 2 Independent Researcher, Calle 6ª, 279 a, Jardines de Vista Alegre, Mérida 97138, Yucatán, Mexico; 3 Instituto Nacional de Investigaciones Forestales, Agrícolas y Pecuarias (INIFAP), Campo Experimental Ixtacuaco, Km 4.5 Carretera Martínez de la Torre-Tlapacoyan, Tlapacoyan 93600, Veracruz, Mexico

**Keywords:** *Carica papaya*, domestication, expression analysis, transcription factors

## Abstract

A genome-wide analysis for two families of key transcription factors (TF; WRKY and NAC) involved in drought response revealed 46 WRKY and 66 NAC members of the *Carica papaya* genome. A phylogenetic analysis grouped the CpWRKY proteins into three groups (I, II a, b, c, d, e and III), while the CpNAC proteins were clustered into 15 groups. The conserved domains, chromosomal localization and promoter cis-acting elements were also analyzed. In addition, from a previous transcriptome study of two contrasting genotypes in response to 14 days of water deficit stress (WDS), we found that 29 of the 46 *CpWRKYs* genes and 25 of the 66 *CpNACs* genes were differentially expressed in response to the WDS. In the present paper, the native wild genotype (WG) (collected in its center of origin) consistently showed a higher expression (transcripts per million; TPM and fold change; FC) than the commercial genotype (CG) in almost all the members of the *CpWRKY* and *CpNAC* gene families. To corroborate this, we selected *CpWRKY50* and *CpNAC83.1* for further evaluation by RT-qPCR. Consistently, the WG showed higher relative expression levels (REL) after 14 days of WDS than the CG, in both the leaves and roots. The results suggest that the CpWRKY and CpNAC TF families are important for drought tolerance in this species. The results may also suggest that, during the domestication process, the ability of the native (wild) *C. papaya* genotypes to respond to drought (including the overexpression of the *CpWRKY* and *CpNAC* genes) was somehow reduced in the current commercial genotypes.

## 1. Introduction

Transcription factors (TFs) are proteins that act in conjunction with various components to control the transcription rate of genetic information from DNA to messenger RNA by binding to cis-acting promoter elements located within the regulatory regions of the target genes [1,2]. Studies have shown that the TF genes are super families that regulate the transcriptional machinery to modulate the target genes expression in a temporal and spatial manner, controlling plant growth and development, the response systems to the terrestrial environment and the physiological processes in response to biotic and abiotic stress [3,4].

WRKY are a family of TF proteins containing DNA-binding domains that have the ability to regulate downstream transcription through a specific recognition of W-box cis-regulatory elements (TTGACT/C) in the promoter regions of the genes related to growth and development, and those involved in the response to biotic and abiotic stress [5]. The WRKY proteins contain a peptide region of 60 amino acids in length and contain one or two highly conserved WRKYGQK motifs that constitute the DNA-binding region with a typical zinc-finger structure [6]. This domain has conserved cysteine and histidine residues attached to a zinc atom with a structure similar to a zinc finger; both are necessary for the binding of the protein to the DNA [6]. In *A. thaliana*, based on the number of WRKY residues and zinc-finger configurations, the WRKYs have been classified into three main groups according to their structure [7]. Group I contains two WRKY domains and one zinc-finger structure classified into I-a and I-b subgroups where the I-a subgroup contains two WRKY domains and a C2H2 structure, while the I-b subgroup has two WRKY domains and a C_2_HC motif [8]. Group II contains one WRKY domain and a C2H2 (Cx4-5Cx22-23HxH) zinc-finger structure. Based on their variants in the zinc-finger motifs, Group II has been subdivided into five subgroups: II-a, II-b, II-c, II-d and II-e. Group III only has one WRKY domain and a C_2_HC motif [6,7]. 

The NAC TF proteins family, on the other hand, is composed of a highly conserved N-terminal DNA-binding domain, a nuclear localization signal sequence and a variable C-terminal domain [9]. The N-terminal region contains a conserved DNA domain of approximately 150 to 160 amino acid residues divided into five conserved subdomains (A–E). The highly conserved subdomains A, C and D play a role in protein stabilization [10]. The nuclear localization signal involved in the NAC recognition process is localized at the C and D subdomains [11,12]. Although the E subdomain shows a lower conservation, it participates with the D domain in a tissue-specific manner. Additionally, the C-terminal region contains a highly variable transcriptional activation region that interacts with the DNA or with the other TFs, as well as some repeated amino acids such as serine, threonine and proline that are enriched in this region [9,10,11,12,13].

The advances in next-generation massive sequencing have allowed for the sequencing of a large number of genomes of different plant species where members of the WRKY and NAC families, among others, seem to play important roles in the abiotic stress (salt, drought, temperature, etc.) tolerance [14]. Thus, it is possible to know the number of WRKYs and NACs present in the genomes of some species, such as *A. thaliana*, *Oriza sativa* and *Glycine max*, among others [15]. However, in *Carica papaya* L., the TF WRKY and NAC have not been classified or characterized. In this context, our group recently published a transcriptomic analysis of *Carica papaya* L. with the aim to study and define whether the wild papaya genotypes (native to Yucatan, its center of origin) differ from the commercial genotypes in terms of their water deficit stress tolerance [16]. The transcriptome study performed in *Carica papaya* L. also helped us to define the possible differences in the expression profiles of these TFs in response to the drought stress between the commercial genotype (cv. Maradol) and the wild genotype of *C. papaya* (native to Yucatan). 

Mexico is one of the main papaya producers in the world, so there are commercial cultivars growing in Yucatán and in another 10 Mexican States with a great commercial value. However, they are rather susceptible to climatic factors, particularly to drought. Among these *C. papaya* cultivars, Maradol cv. is the main commercial genotype grown in the country and is in fact exported to the USA and other countries due to its fruit quality and nutritional and organoleptic characteristics. However, their intensive cultivation has been associated with a further loss of diversity of this species.

*C. papaya* L. is considered a native species in southeastern Mexico, with Veracruz, Tabasco and the Yucatan Peninsula as the sites where the greatest diversity of this species is found [17,18]. Like any fruit tree, the papaya demand large amounts of water to increase the quantity and quality of the harvested products [19]. However, over the last decade, global losses in crop production due to drought was estimated at $30 billion dollars. The water demand for agriculture could double by 2050, but freshwater availability is projected to decline by 50% due to climate change [20]. Therefore, there is a need to develop new varieties with increased drought tolerance or increased water use efficiency to reduce the negative effects of drought on the papaya production.

Wild papayas may represent a source of genes for abiotic stress tolerance as they have shown the ability to detect water deficits and develop strategies to counteract water stress [18]. The wild papaya genotypes may have developed the ability to maintain their biological functions, even under low water potentials, turning them, by definition, into drought tolerant genotypes [21].

It has been hypothesized, that the susceptibility of the commercial genotype to WDS could be related to the loss of key transcription factor genes in this genotype during its domestication process. In contrast, the drought stress tolerance presented by the wild *C. papaya* genotype, collected in undisturbed sites in the Yucatan Peninsula, may have conserved its ability to overexpress the *CpWRKY* and *CpNAC* genes in response to WDS, which are key to attenuate the abiotic stresses damage and to mitigate the losses in productivity caused by climate change.

Therefore, the aim of the present study was to identify and characterize all the members of the CpWRKY and CpNAC transcription factor families in the *C. papaya* genome, and to analyze which genes of those TF families were differentially expressed in two contrasting genotypes (a commercial genotype “Maradol” and a wild genotype collected in Yucatan) when the plants were subjected to 14 d of water deficit stress, based on a previous transcriptomic study. Finally, to corroborate whether the wild genotype might show a higher expression than the commercial genotype in response to drought, we used RT-qPCR studies with a gene selected from both the CpWRKY and CpNAC TF families.

## 2. Results

### 2.1. Behavior of Both C. papaya Genotypes in Response to Water Deficit Stress (WDS)

**Figure 1:**
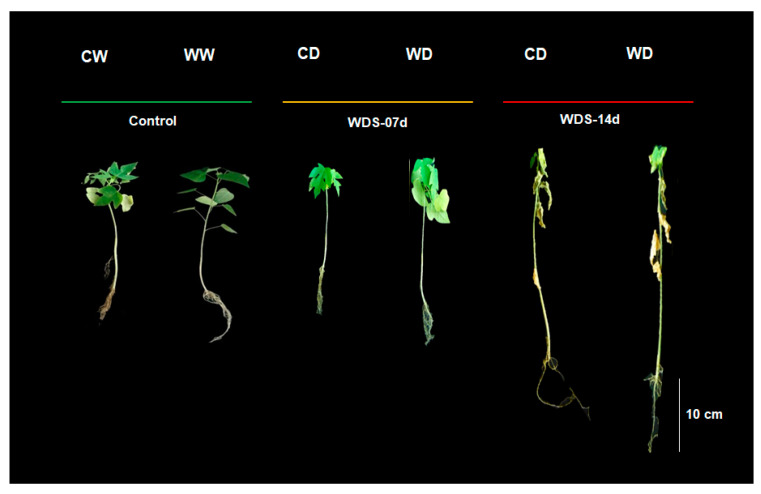
Images of *Carica papaya* L. plants from both genotypes (commercial genotype; C and wild genotype; W). *Carica papaya* plants under optimal watering conditions (CW and WW) and after 7 (WDS-07d) and 14 days (WDS-14d) of water deficit stress (CD and WD). Green line indicates well-watered controls; yellow line indicates mild WDS; red line indicates severe WDS.

The well-watered control plants from both genotypes showed green and turgid leaves. After 7 days of WDS, the commercial genotype (CG) lost some of its leaves and the remaining leaves appeared less turgid while the wild genotype (WG) maintained more leaves and they appeared more turgid. After 14 d of WDS, the CG plants lost most of their leaves and they appeared wilted, while the plants from the WG maintained more leaves and some leaves were still green and appeared more turgid. The WG also showed less root damage than the CG after being exposed to 7 and 14 d of WDS (Figure 1).

### 2.2. Genome-Wide Identification of the WRKY and NAC Gene Family in C. papaya

A total of 46 putative full-length WRKY genes were identified in the *C. papaya* L. genome. All the CpWRKY sequences showed a high identity (above 60%) in relation to the pairwise comparison analysis of the predicted proteins sequences of the AtWRKY family from *A. thaliana*. In particular, the evm.model.supercontig_12.58 (CpWRKY2), evm.model.supercontig_152.36 (CpWRKY6), evm.model.supercontig_768.1 (CpWRKY12), evm.model.supercontig_2011.1 (CpWRKY14), evm.model.supercontig_52.143 (*CpWRKY21*) and evm.model.supercontig_87.103 (CpWRKY31) showed a high similarity (above 95%) to their *A. thaliana* counterparts (Supplementary Table S1).

In the case of the NAC family, a total of 66 NAC genes were identified in *C. papaya* L. However, these CpNAC sequences showed an identity percentage ranging from 31 to 97.59%. The pairwise comparison analysis of the predicted CpNAC proteins sequences indicated that the evm.model.supercontig_29.118 (CpNAC26), evm.TU.contig_29561.1 (CpNAC57), evm.model.supercontig_78.40 (CpNAC75), evm.model.supercontig_64.115 (CpNAC78) and evm.model.supercontig_111.22 (CpNAC100) sequences showed a high similarity (above 90 %) to their *A. thaliana* counterparts (Supplementary Table S2).

### 2.3. Phylogenetic Analysis and Classification of the WRKY and NAC Proteins between A. thaliana and C. papaya

**Figure 2:**
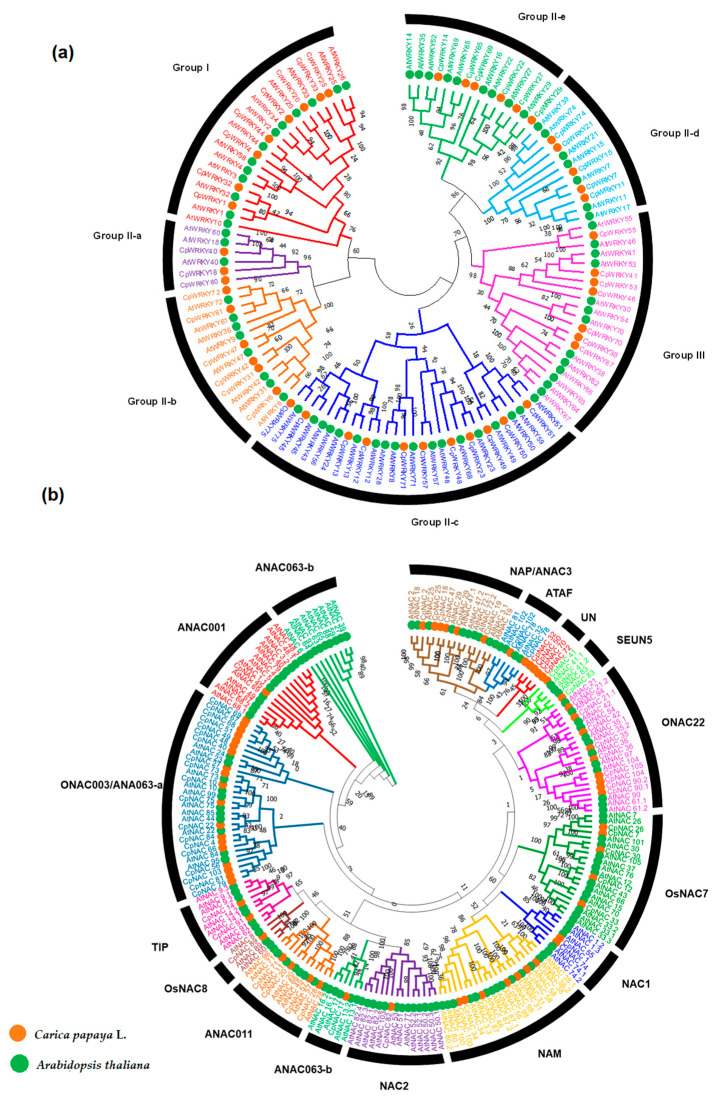
Unrooted phylogenetic trees of *Carica papaya* L. (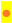) and *Arabidopsis thaliana* (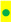) of the WRKY and NAC proteins. (**a**) Phylogenetic tree of WRKY domain protein from *A. thaliana* and *C. papaya* L. The subgroups classified as I, IIa, IIb, IIc, IId, IIe and III are represented. (**b**) Phylogenetic tree of the NAC (NAP/ANAC3, ATAF, UN, SEUN5, ONAC22, OsNAC7, NAC1, NAM, NAC2, ANAC063-b, ANAC011, OsNAC8, TIP, ONAC003/ANAC063-a, ANAC001 and ANAC063-b) domain protein from *A. thaliana* and *C. papaya* L. The 15 subgroups are distinguished by different colors. Both trees were aligned with MEGA 11 software based on the UPGMA method using the Jones–Taylor–Thornton (JTT) substitution model to generate the phylogenetic trees using the neighbor-joining method with 10,000 bootstrap replicates.

The phylogenetic analysis of the WRKY proteins performed between the *A. thaliana* and *C. papaya* genomes indicated that the 46 CpWRKYs formed three groups (I, II and III) that in turn form seven subgroups, as Subgroup II was sub-classified as Group II a, b, c, d and e. Both genomes showed at least three genes in each subclade (Figure 2a). Group I was formed by eight proteins: CpWRKY1, CpWRKY2, CpWRKY4, CpWRKY20, CpWRKY25, CpWRKY32, CpWRKY33 and CpWRKY44, while Group III was formed by seven proteins: CpWRKY38, CpWRKY41, CpWRKY46, CpWRKY53, CpWRKY55, CpWRKY67 and CpWRKY70. Group II was divided into three proteins for the II-a subgroup, six for the II-b subgroup, 11 for the II-c subgroup, five for the II-d subgroup and six for the II-e subgroup (Figure 2a). The phylogenetic analyses also showed that the WRKY proteins from the two species were clustered in pairs, except in the case of WRKY25, indicating that they were orthologous WRKY domains from the same lineage.

With respect to the NAC family, the CpNAC and AtNAC proteins were clustered together and, according to their homology, the CpNAC and AtNAC proteins were classified into 15 subgroups: ATAF, NAP/ANAC3, SEUN5, ONAC22, OsNAC7, NAC1, NAM, NAC2, ANAC063-b, ANAC011, OsNAC8, TIP, ANAC001, ONAC003/ANAC063-a, ANAC63 and an unclassified group (UN) (Figure 2b). The results indicated that at least one CpNAC protein was classified within each subgroup. Among these subgroups, the unknown members of the subgroup (not present in *A. thaliana*) had the largest number of CpNAC proteins (12), followed by NAP/ANAC3 with nine proteins and ONAC022 and NAM with seven proteins each. The ANAC001, ANAC063-b, NAC1, SEUN5 and TIP had two CpNAC proteins. The OsSNAC8, ANAC63 and NAC2 subgroups had the fewest members, with only one CpNAC protein (Figure 2b).

### 2.4. Multiple Sequence Alignment of the CpWRKY and CpNAC Proteins

The multiple sequence alignment of the WRKY domains between *C. papaya* and *A. thaliana* showed that this family spanned approximately 60 amino acids. Additionally, it showed the presence of a WRKYGQK conserved domain at the N-terminal with a C2H2-type zinc-finger motif at the C-terminal end (Supplementary Figure S1). Group I of CpWRKY proteins contained two WRKY domains (WRKYGQK) and two C2H2-type zinc-finger motifs in their N-terminal and C-terminal. Groups II and III contained one WRKY domain and a C2H2-type zinc-finger motif. Interestingly, in Group I, only CpWRKY20 was defined by one WRKYGK and a C2H2-type zinc-finger motif in the C-terminal. Furthermore, all seven members in Group III contained the conserved WRKYGQK domain and the special C2HC-type zinc fingers (Supplementary Figure S2). Thus, the conserved heptapeptide WRKYGQK characteristic of this family recognized the W-box to promote the expression of the target genes. The Heptapeptide WRKYGQK in most CpWRKY proteins was highly conserved. However, in CpWRKY50 and CpWRKY51, it mutated into WRKYGKK, although the same mutation was observed in the *A. thaliana* AtWRKY50 and AtWRKY51 members.

In the case of NAC, the homology alignment for the NAC domains between *C. papaya* and *A. thaliana* showed that all the CpNACs contained a NAC conserved domain. Additionally, the NAC conserved domains at the N-terminal were divided into five conserved subdomains (A–E). The NAC domain contained nuclear localization signals (NLS) and DNA-binding domains (DBD) (NLS/DBD) (Supplementary Figure S3). The CpNACs showed a C-terminal region with a relatively divergent transcriptional activation region (TAR) and a transmembrane motif (TMM). However, the C-terminal regions in *C. papaya*, showed no significant similarity with the NAC family of *A. thaliana* (Supplementary Figure S3).

### 2.5. Conserved Motifs of CpWRKY and CpNAC Proteins

**Figure 3:**
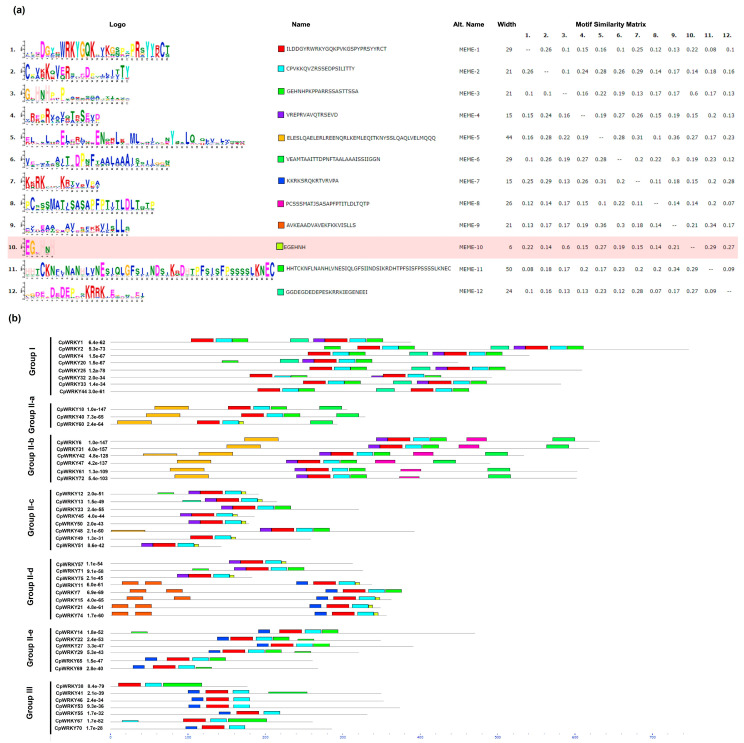
Conserved motifs distribution of the CpWRKYs proteins. (**a**) Sequence logo view of the consensus of the CpWRKYs domain sequences. The height of the letter (amino acid) at each position represents the degree of conservation in each of the 12 predicted motifs (motif 1–12). (**b**) Visualization of the classification of the CpWRKYs proteins and the distribution of the predicted motifs. The conserved motifs were detected using the Multiple EM for Motif Elicitation (MEME) software. Each motif with conserved amino acid residues is represented with different colors.

**Figure 4:**
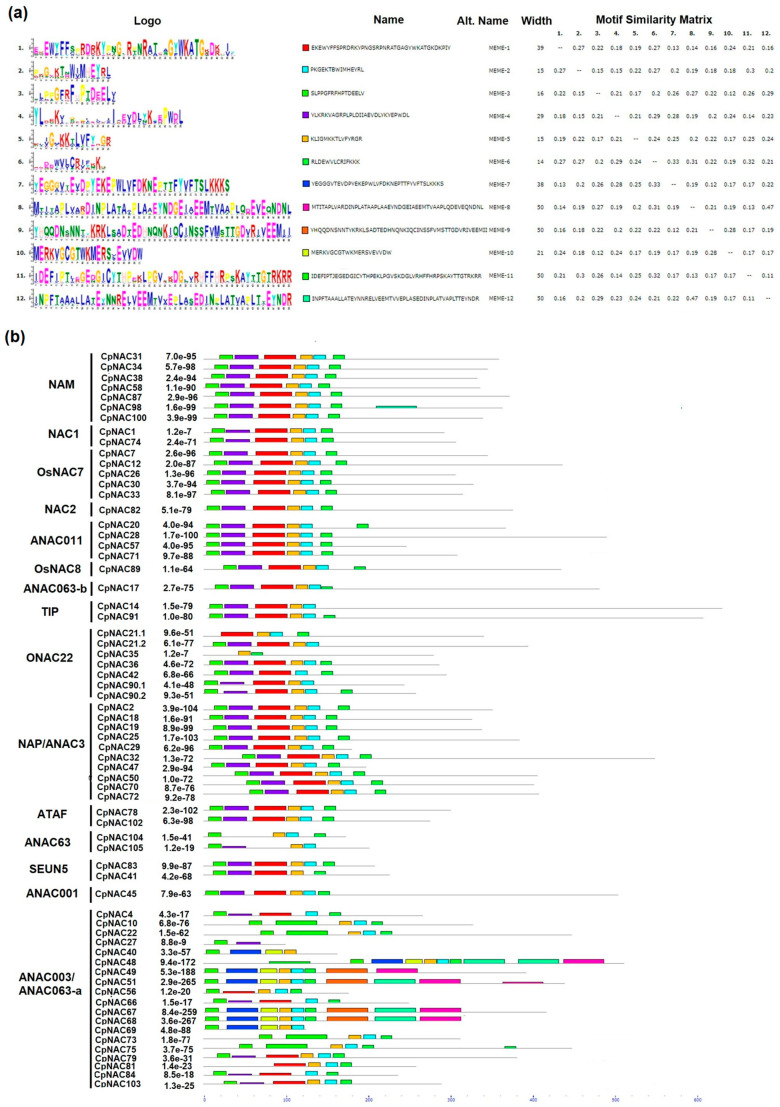
Conserved motifs distribution of the CpNACs proteins. (**a**) Sequence logo view of the consensus of the CpNACs domain sequences. The height of the letter (amino acid) at each position represents the degree of conservation in each of the 12 predicted motifs (motif 1–12). (**b**) Visualization of the classification of the CpNACs proteins and the distribution of the predicted motifs. The conserved motifs were detected using the Multiple EM for Motif Elicitation (MEME) software. Each motif with conserved amino acid residues is represented with different colors.

Our analysis, using the Multiple Expectation Maximization for Motif Elicitation online program, revealed 12 conserved motifs for CpWRKY (Figure 3) and 12 conserved motifs for the CpNAC (Figure 4) proteins in *C. papaya*. The conserved motifs analysis showed that each of the 46 CpWRKY protein sequences showed an E-value less than 10, and the motif matches shown had a position *p*-value lower than 0.0001. Likewise, in the CpWRKY protein sequences, the 10 EGEHNH motif (with a pale red background) was very similar to other earlier specified motifs (Figure 3a). Our results showed a highly conserved WRKY domain in all the CpWRKYs, thus all the CpWRKYs contained WRKY motif 1 and C2HC-type zinc-finger motifs 2 and 3 (Figure 3a). The CpWRKY proteins clustered in the same groups and subgroups that shared a similar motif composition indicated functional similarities among members of the same subgroup, but differences in the motif patterns among members of the same subgroup (Figure 3b).

In terms of the CpNAC proteins, our analysis revealed 12 conserved motifs, and their distributions by classes are shown in Figure 4a. Most of the CpNACs contained at least one of the five main motifs (motifs 1, 2, 3, 4 and 5) that were annotated as a NAC domain and/or no apical meristem (NAM) domain. Therefore, all the CpNAC proteins identified in this study have conserved features of the NAC family. Notably, the motifs in subgroup ANAC063 were the most diverse, which corresponds to the intron/exon distribution. Additionally, the CpNAC proteins clustered in the same subgroups shared a similar motif composition. Therefore, these proteins may have functional similarities among members of the same subgroup, however, differences in the motif patterns among members of the same subgroup were also observed (Figure 4b).

### 2.6. Cis-Elements Analysis of CpWRKYs and CpNACs Genes

**Figure 5:**
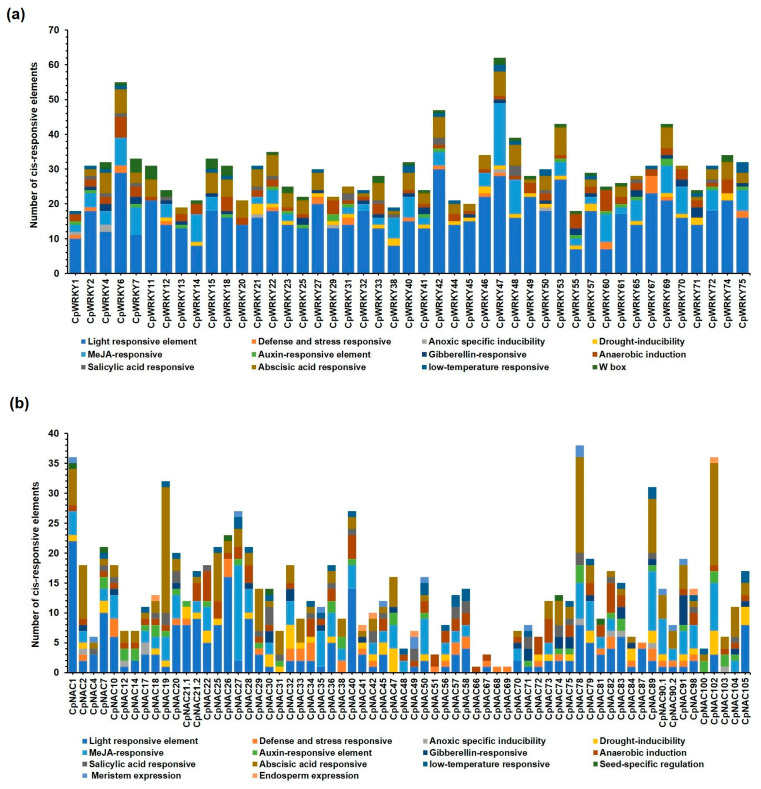
In-silico analysis of the cis-acting regulatory elements present in the promoter regions (2.0 kb upstream) of the (**a**) 46 CpWRKYs and (**b**) 66 CpNACs genes families using the PlantCARE software. The number of cis-responsive elements induced by each stress condition (drought, low temperature, anaerobic, etc.) or by plant hormones (ABA, MeJA, SA, GA, auxins), are shown in different colors.

The cis-acting elements of all 46 *CpWRKYs* and 66 *CpNACs* gene families were predicted using the Plant CARE online analysis website. In the case of *CpWRKYs*, we identified the cis-acting regulatory elements that were responsive to light, stress condition (defense and stress responsive), inducible by anoxia, drought, anaerobic conditions, or responsive to low-temperature responsiveness and W-box. Additionally, the cis-elements were identified as being responsive to plant hormones such as MeJA, auxin, gibberellin, salicylic acid and abscisic acid (Figure 5a). 

The 45 CpWRKY genes had light responsive cis-elements, 40 of them had cis-elements inducible by anaerobiosis, 39 had ABA-responsive elements (ABRE), 31 had MeJA-responsive elements (CGTCA motif), 26 had drought-inducible cis-elements (MYB transcription factor binding site involved in drought inducibility), 26 had a W-box (WRKY transcription responsive), 23 had gibberellin-responsive elements, 20 had auxin-responsive elements, 15 had salicylic-acid-responsive elements (TCA element), 15 of them had defense and stress-responsive elements and only six had cis-elements inducible by anoxia. CpWRKY47, CpWRKY6 and CpWRKY42 were the *CpWRKYs* that showed the highest number of total cis-elements with 62, 55 and 47, respectively. On the contrary, those showing the lowest number of cis-elements were CpWRKY13, CpWRKY38 and CpWRKY1 (with 19, 19 and 18 cis-elements, respectively) (Figure 5a).

In the case of the CpNACs, except for the W-box element, the same cis-acting elements found in the CpWRKYs were identified in this TF family with the addition of seed-specific regulation, meristem expression and endosperm expression cis-elements (Figure 5b). In total, 55 CpNAC genes showed light-responsive cis-elements, 49 had ABA-responsive elements, 46 had cis-elements by anaerobic induction, 39 showed MeJA-responsive cis-elements, 33 had cis-elements inducible to drought, 30 had defense and stress-responsive cis-elements and 29 of them had auxin-responsive cis-elements. Interestingly, CpNAC20 showed MeJA, auxin, gibberellin, salicylic acid and abscisic-acid-responsive cis-elements. On the other hand, some CpNACs showed cis-elements related to plant development, one showed meristem expression, six showed seed-specific regulation and six showed endosperm-expression-responsive cis-elements. Likewise, CpNAC78, CpNAC1, CpNAC102, CpNAC19 and CpNAC89 showed the highest number of total cis-elements (38, 36 and 36 cis-elements, respectively). On the contrary, CpNAC66, CpNAC68 and CpNAC69 showed only one cis-element (Figure 5b).

### 2.7. Chromosomal Lacalization of CpWRKY and CpNAC Genes

**Figure 6:**
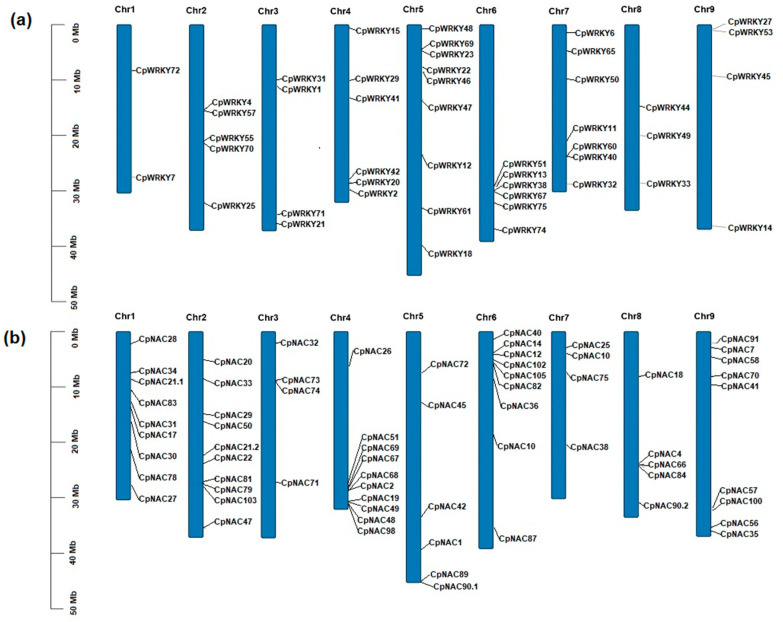
Chromosomal localization of the (**a**) 46 *CpWRKY* and (**b**) 66 *CpNAC* genes along the nine chromosomes of *C. papaya* genome. The number of each chromosome is given at the top of each chromosome and, at the left axis, the approximate physical location of each *CpWRKY* and *CpNAC* genes are shown. The above results were visualized using TBtools [22].

At least one of the 46 *CpWRKY* genes was present in each of the nine chromosomes. Chromosome 5 hosted the largest number of *CpWRKY* genes with nine, followed by chromosomes 6 and 7 with seven *CpWRKY* genes. It is noteworthy that in chromosome 6, six *CpWRKY* genes were clustered together (*CpWRKY57*, *CpWRKY51*, *CpWRKY13*, *CpWRKY38*, *CpWRKY67* and *CpWRKY75*). On the other hand, chromosome 1 hosted the lowest number of CpWRKY genes, with only two, followed by chromosome 8 with three (Figure 6a).

Regarding the *CpNAC* genes, at least one of the 66 *CpNAC* genes was present in each of the nine chromosomes. Chromosomes 2 and 4 hosted the largest number of *CpNAC* genes with 10, followed by chromosomes 1, 6 and 9 with nine CpNAC genes. It is noteworthy, that in chromosome 4, nine *CpNAC* genes were clustered together (*CpNAC51*, *CpNAC69*, *CpNAC67*, *CpNAC68*, *CpNAC2*, *CpNAC19*, *CpNAC49*, *CpNAC48* and *CpNAC98*). Additionally, in chromosome 6, six *CpNAC* genes were clustered together (*CpNAC14*, *CpNAC12*, *CpNAC102*, *CpNAC105*, *CpNAC82* and *CpNAC40*). On the other hand, chromosomes 3 and 7 hosted the lowest number of *CpNAC* genes with four, followed by chromosome 8 with five (Figure 6b).

### 2.8. TPM and FC of the CpWRKYs and CpNACs Genes under Water Stress Obtained from RNA-Seq Data

The analysis of our previous transcriptomic study of exposure to a water deficit [16] revealed 29 differentially expressed *CpWRKY* genes in terms of transcripts per million (TPM). In order to name the 29 WRKY-type contigs identified in the transcriptome, a pairwise identity percentage analysis was performed between the 46 CpWRKY found in the genome against the 29 WRKY contigs identified in the transcriptome (Supplementary Table S3). In the case of the *CpNAC* genes, a pairwise identity percentage was performed between the 66 *CpNAC* genes found in the genome, against the 25 *CpNAC* contigs identified in the transcriptome (Supplementary Table S4). 

**Figure 7:**
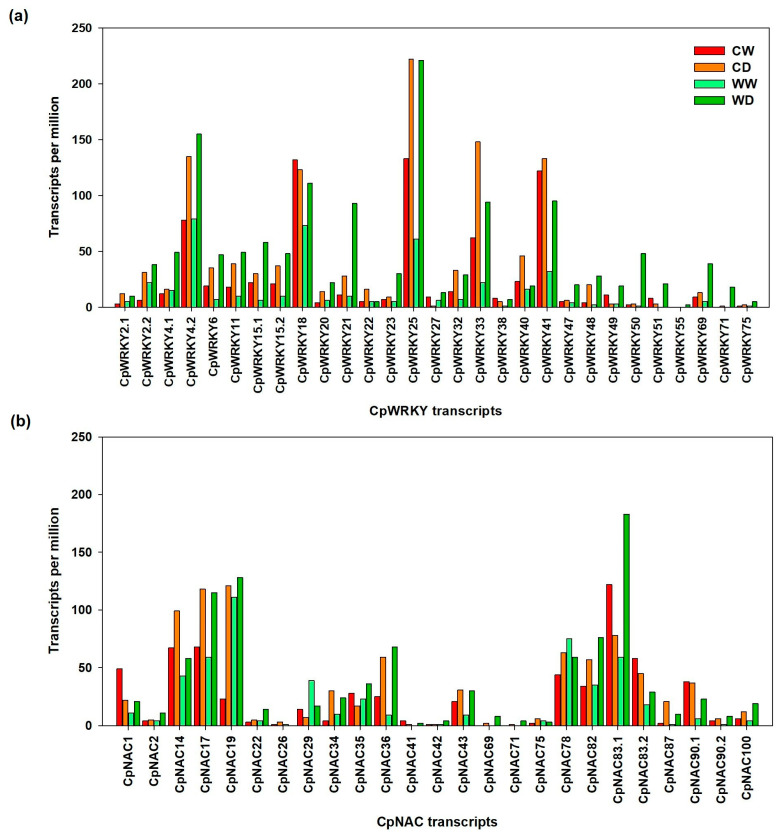
Number of transcripts per million (TPM) found in the commercial (C) and wild (W) papaya genotypes from a transcriptomic study on *C. papaya* plants exposed to WDS for 14 d. (**a**) The TPM of the 29 *CpWRKY* members and (**b**) the TPM of the 25 *CpNAC* members when well-watered (CW and WW) and when subjected to 14 of WDS (CD and WD).

In terms of the TPM for the *CpWRKY* genes under well-watered conditions, the wild genotype (WW) showed a lower TPM than the commercial cultivar (CW) in *CpWRKY6*, *CpWRKY11*, *CpWRKY25*, *CpWRKY33*, *CpWRKY41* and *CpWRKY69*. However, under water deficit conditions (WDS), the wild genotype (WG) showed a higher TPM than the commercial genotype (CD) in most *CpWRKY* genes, except in *CpWRKY25*, *CpWRKY33*, *CpWRKY40* and *CpWRKY41* (Figure 7a). 

In the case of the TPM for the *CpNAC* genes under well-watered conditions, the wild genotype (WW) showed a lower TPM than the commercial genotype (CW) in most CpNACs members. However, under WDS, the wild genotype (WD) showed a higher TPM than the commercial cultivar (CD) in many *CpNAC* genes, in particular, *CpNAC83.1* (183 TPM) and *CpNAC19* (128 TPM). However, in *CpNAC14*, *CpNAC17*, *CpNAC34*, *CpNAC43* and *CpNAC78*, the wild genotype showed a lower TPM than the commercial cultivar, even under WDS conditions (Figure 7b).

**Figure 8:**
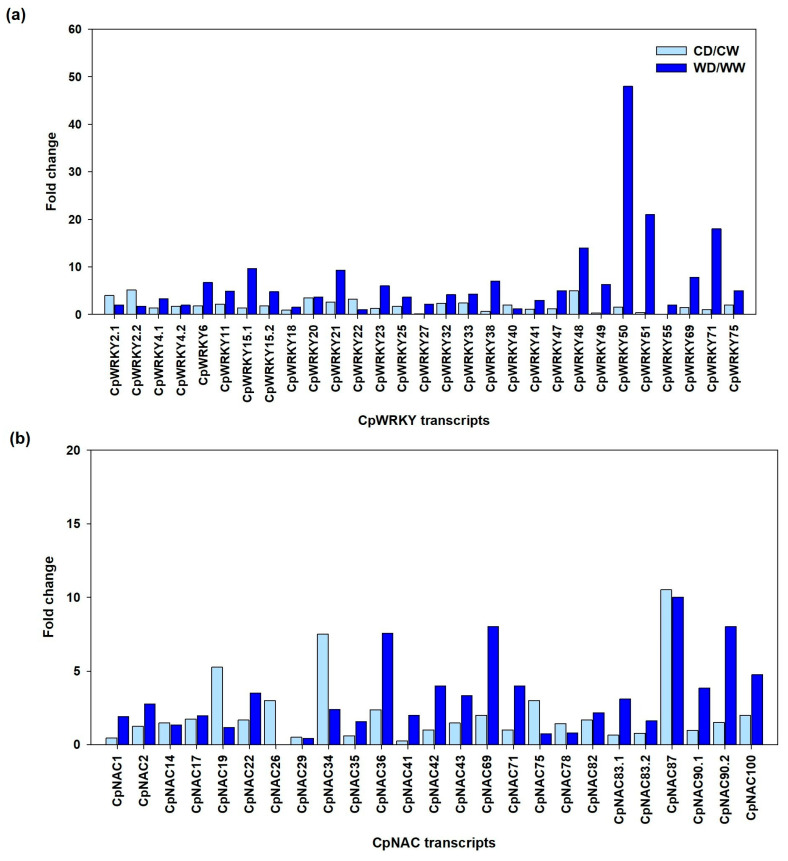
Fold change (FC) in the gene expression of (**a**) the 29 *CpWRKY* members and (**b**) the 25 *CpNAC* members found in *C. papaya* plants exposed to 14 d of water deficit stress (CD or WD) relative to well-watered conditions (CW or WW) in both commercial (CD/CW) and wild (WD/WW) papaya genotypes.

Hence, in response to the water deficit, most *CpWRKYs* and *CpNACs* from the wild genotype (tolerant) had a higher expression (in terms of TPM) than those from the commercial genotype (susceptible), suggesting that these CpWRKYs and CpNACs might be related to the drought tolerance in papaya. Regarding the fold change (FC) values that indicate how many times a gene was expressed in response to WDS relative to its own expression when well-watered, again the wild genotype showed much higher FC values (WD/WW) in most CpWRKYs than the commercial genotype (CD/CW). In particular, the wild genotype showed the highest FC values for *CpWRKY50*, *CpWRKY51*, *CpWRKY71* and *CpWRKY48* with FC values of 48, 21, 18 and 14, respectively. Only in a few cases, the commercial genotype showed slightly higher FC values than the wild genotype in *CpWRKY 2.1*, *CpWRKY2.2*, *CpWRKY22* and *CpWRKY40* (Figure 8a). 

In the case of most *CpNACs*, the wild genotype also showed higher FC values than the commercial genotype, in particular *CpNAC87* (FC of 10), *CpNAC69* (FC of 8), *CpNAC90.2* (FC of 8), *CpNAC36* (FC of 7.5), *CpNAC100* (FC of 4.7), *CpNAC42* (FC of 4) and *CpNAC83.1* (FC of 3.1). However, in some *CpNACs*, the commercial genotype showed higher FC values (CD/CW) than the wild genotype (WD/WW), for instance, in *CpNAC87* (FC of 10.5), *CpNAC34* (FC of 7.5), *CpNAC19* (FC of 5), *CpNAC75*, *CpNAC26* and *CpNAC78* (Figure 8b). 

Therefore, based on their TPM and FC values, *CpWRKY50* (with 48 TPM and 48 FC) and *CpNAC83.1* (with 183 TPM and 3.16 FC), were selected for further RT-qPCR analysis at different times and different papaya tissues (leaves and roots).

### 2.9. Relative Expression Levels of the CpWRKY50 and CpNAC83.1 Genes Associated to the WDS Tolerance

**Figure 9:**
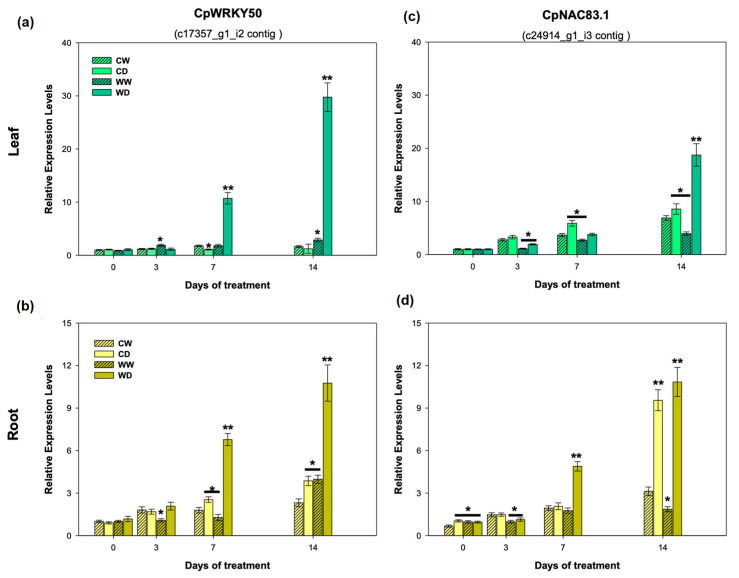
Relative expression levels (REL) found in the different tissues of two contrasting *C. papaya* genotypes estimated by RT-qPCR. The REL values for *CpWRKY50* (**a**,**b**) and for *CpNAC83.1* (**c**,**d**) found in the leaves (**a**,**c**) and roots (**b**,**d**) from the commercial genotype (CW) and from the wild genotype (WW) under irrigated conditions, and from the commercial genotype (CD) and the wild genotype (WD) under water deficit stress (WDS) conditions, after 0, 3, 7 and 14 d of treatment (* *p* < 0.05 and ** *p* < 0.001).

These two candidate genes (*CpWRKY50* and *CpNAC83.1*) were validated through the RT-qPCR analysis. The results confirmed that under optimal watering conditions (day 0 and WW) *CpWRKY50* showed low relative expression levels (REL) in the leaves and roots of both genotypes (Figure 9a,b). However, at 7 and 14 days of water deficit stress (WDS), this gene showed the highest REL in the leaves of the wild genotype (WD) (Figure 9a). The exposure to WDS also induced a significant increase in the expression of the *CpWRKY50* gene in the roots of the wild genotype after just 7 days of WDS and it increased further at day 14 (Figure 9b).

For the *CpNAC83.1* gene under irrigation conditions, the leaves showed a very low expression in both genotypes (WW and CW) (Figure 9c). However, from day 3 to 14, the expression levels of the *CpNAC83.1* gene increased significantly in the wild genotype (WD) that showed the highest REL values and even doubled its expression when compared to the expression reached in the commercial genotype exposed to WDS (CD) (Figure 9c). In the root tissue at day 7, the wild genotype (WD) reached significantly higher REL values than the commercial one (CD). However, at day 14 under the WDS conditions, both genotypes showed significantly higher REL values for the *CpNAC83.1* gene than those found under the well-watered conditions (Figure 9d). 

## 3. Discussion

Water deficit stress causes a reduction in the physiological performance (reduced stomatal conductance, reduced photosynthesis, reduced Fv/Fm values, etc.) in young plants of the commercial cultivar of papaya [16,23]. It is believed that some TFs such as WRKY may play important roles in triggering the response mechanisms to counteract the negative effects of drought in plants (14). Therefore, the study of the structure, phylogeny and expression profiles of these TFs in response to WDS is important to set the basis for the future development of new varieties with an enhanced ability to cope with this abiotic stress.

In the present study, our analysis revealed 46 CpWRKY and 66 Cp NAC members in the *C. papaya* genome, while in *A. thaliana* we confirmed 71 AtWRKY and 132 AtNAC members. This is consistent with our findings in the other TF families of CpTGA, CpSHINE, CPMYB and CpHSF, confirming that the papaya showed fewer members in each TF family than those found in *A. thaliana* [16,23,24]. This is also in agreement with the reports from whole genome sequencing studies in *C. papaya* performed by [25,26] where the number of WRKY and NAC genes in *C. papaya* showed fewer members than those reported in *A. thaliana* or *O. sativa.* Other high-throughput sequencing studies have reported the number of WRKY members present in some species such as rice (103), soybean (197) and pineapple (54) [15]. This reduction in the number of members in the TF families found in *C. papaya* has been attributed to the lack of the genome duplication in *C. papaya* that did occur in the *A. thaliana* genome, as suggested by Ming et al. [25].

Our own phylogenetic analysis of the WRKY and NAC families present in the *C. papaya* and *A. thaliana* genomes showed that the 46 CpWRKY proteins were grouped in three different groups (I, II, II), although Group II was subdivided into a, b, c, d and e. Likewise, the 66 CpNACs proteins were clustered into 15 subgroups [27]. In all the subgroups from both TF families found in *A. thaliana*, we found at least one TF member present in the *C. papaya* genome, which may indicate some degree of redundancy in each group in the case of *A. thaliana* [6,7]. 

However, there were 12 CpNAC genes in papaya that did not have a counterpart in the *A. thaliana* genome (but they showed the ONAC003/ANAC063-a motifs). These 12 genes found in papaya merit further investigation as they might be novel NAC genes related to WDS in tropical plants. Clearly, more research must be undertaken to clarify this possibility.

Our analysis of the conserved domains showed that all the 46 CpWRK sequences found in the genome have the characteristic domains of this TF family. They showed 12 characteristic motifs, and all the CpWRKY sequences were grouped according to their motifs in Groups I, II (a, b, c, d or e) or III. Likewise, all 66 CpNAC sequences found in the *C. papaya* genome showed the characteristic conserved domains of this TF family. At least the 1st, 2nd, 3rd, 4th or 5th motifs were observed in all the CpNAC sequences. It is noteworthy that the sequences CpWRKY 50 and CpWRKY 51 showed a mutation in one of the motifs of the domain WRKY (WRKYGKK instead of WRKYGQK), which was not present in any of the other 44 CpWRK sequences. However, this mutation was also found in the AtWRK50 and AtWRKY51 genes in the *A. thaliana* genome. 

The present analysis of the promoter cis-acting elements found in the genome revealed that all 46 genes (except one sequence) presented light-responsive elements. Interestingly, 39 CpWRKY members had abscisic-acid-responsive elements (with AACCCGG and ACGTG, ABRE motifs), 31 CpWRKY members had MeJA-responsive elements (with CGTCA and TGACG motifs), 20 CpWRKY members showed auxin-responsive elements (with GGTCCAT, AuxRR core motif) and 26 CpWRKY members showed drought-inducible elements (with CAACTG, MBS motif). The other nine CpWRKY members showed the cis-regulatory elements involved in endosperm expression (with TGAGTCA, GCN4 motif).

In the CpNAC TF family, 93.9% of the genes showed light-responsive elements, 50 genes showed ABA-responsive elements, 49 genes (with ACGTG and CACGTG) showed ABRE motifs, while another 9 genes showed responsiveness to ABA, 40 showed MeJA-responsive cis-elements (with TGACG and CGTCA motifs) and 30 NAC genes showed defense and stress-responsive cis-elements (with ATTCTCTAAC and GTTTTCTTAC, TC-rich repeats). Interestingly, 40 CpNAC showed MYB binding, either site-light or drought-inducible cis-elements (with AACCTAA, MRE and CAACTG, MBS motifs, respectively). 

Our data indicated that most members from the CpWRKY and CpNAC TF families were involved in drought or other stress-responsive activities, and most of them had ABA-responsive elements.

From our previous transcriptome study (16), we found that 29 of the 46 transcripts of *CpWRKY* and 25 of the 66 transcripts of *CpNAC* were differentially expressed in response to 14 d of WDS. Almost all the members of *CpWRKY* (20 of 29 genes) and *CpNAC* (14 of 25) increased their expression under drought stress. 

Under well-watered conditions, most *CpWRKY* members from the wild genotype showed lower expression levels (in terms of TPM) than the commercial genotype. On the contrary, under WDS conditions, most *CpWRKY* members from the wild genotype showed consistently higher expression levels than the commercial genotype. For instance, *CpWRKY25* in the wild genotype increased from 61 TPM under WW conditions to 221 TPM under WDS conditions. 

In the case of *CpNAC* under WW conditions, various *CpNAC* members in the wild genotype showed lower expression levels than the commercial genotype, but, under WDS, those genes showed a higher expression in the wild genotype than in the commercial genotype. For instance, *CpNAC83.1* in the wild genotype showed a particularly high TPM in response to WDS, increasing from 59 under WW conditions to 183 TPM under WDS conditions. 

In terms of the fold change (FC), the wild genotype showed higher FC values (FC values ranging from 9.7 to 25) than the commercial genotype (FC values ranging from two to five) in almost all (25 of 29) *CpWRKY* genes. *CpWRKY50* and *CpWRK51* showed particularly high FC values (48 and 21, respectively) in the wild genotype. It is interesting that the CpWRKY sequences that showed a mutation in one of the motifs (CpWRKY50 and CpWRKY51) were also those that had the highest gene expression (in terms of the fold change) of all the other *CpWRKY* members after 14 d of WDS. Although it requires further investigation, this is certainly an interesting correlation, and it may provide information on the relative relevance of these domains and motifs on the drought tolerance mechanisms of this species. Our results confirmed previous reports of the *WRKY* genes in papaya where they report that the FT43.76 and FT5.242 genes were expressed under the drought treatment [28] genes that in fact correspond to our *CpWRKY75* and *CpWRKY33* genes, which were also expressed under our WDS treatment. 

In the case of the *CpNAC* TF family, the wild genotype also showed higher FC values (ranging from 1.9 to 8 values) than the commercial genotype (FC ranging from 0.5 to 10.5 values) in almost all (17 of 25) *CpNAC* genes. 

To further corroborate this, based on their high number of TPM and high FC values shown by the wild (tolerant) genotype, we selected one gene from the *CpWRKY* family (*CpWRKY50*) and one from the *CpNAC* family (*CpNAC83.1*) for further evaluation using RT-qPCR. Consistently, after 7 and 14 days of water deficit stress, both genes (*CpWRKY50* and *CpNAC83.1*) showed higher RT-qPCR expression values (REL) in the wild genotype than in the commercial one, in both the leaves and roots.

Our data suggest that the higher drought tolerance capacity of the papaya wild genotype is at least partly related to its capacity to overexpress the *CpWRKY* and *CpNAC* genes in response to drought. It is interesting that, under well-watered conditions, both genotypes showed a low expression in the *CpWRKY* and *CpNAC* genes, but when water was limited, the commercial genotype showed only a slight increase in the expression of these genes, while the wild genotype was capable of increasing the expression of these genes (*CpWRKYs* and *CpNACs*) in a significant manner. 

Other studies of the papaya have shown that, during domestication, changes in the plant height, fruit size and fruit color certainly occurred, showing an intermediate fruit phenotype between the wild and cultivated plants [17,29]. This may suggest that during the domestication process (domestication syndrome), the capacity to respond to water deficits by expressing most of the family members of the *CpWRKY* and *CpNAC* TF families shown by the native wild genotype was somehow reduced in the current commercial genotype. The results also indicate that *CpWRKY50* and *CpNAC83.1* might be good candidate genes for the transformation of drought susceptible genotypes in breeding programs aiming to increase the drought tolerance in this species.

## 4. Materials and Methods

### 4.1. Identification of the WRKY and NAC Sequences, and the Gene Structure

The WRKY and NAC protein sequences were obtained from the Plant Transcription Factor Database V4.0 (PlnTFDB 4.0)—Universitaet Potsdam (http://plntfdb.bio.uni-potsdam.de/ Accessed on 18 July 2022) and the Plant Transcription Factor database V5.0 (PlantTFDB 5.0)—Center for Bioinformatics, Peking University (http://planttfdb.cbi.pku.edu.cn/index.php Accessed on 18 July 2022). The information of both databases was verified performing TBLASTX using the WRKY and NAC sequences of *A. thaliana* against *C. papaya*. The genomic sequences, ID numbers and coding sequences (CDS) corresponding to each predicted CpWRKY and CpNAC gene were obtained from the Phytozome database 10.1 (https://phytozome.jgi.doe.gov/pz/portal.html Accessed on 25 July 2022). The SMART online software (http://smart.embl-heidelberg.de/ Accessed on 25 July 2022) and InterProScan tool (http://www.ebi.ac.uk/Tools/pfa/iprscan/ Accessed on 25 July 2022) were used to identify the integrated domains in putative papaya WRKY and NAC proteins. A pairwise identity was performed to assign the CpWRKY and CpNAC proteins.

### 4.2. Multiple Sequence Alignment, Phylogenetic Analysis and Motif Identification

Multiple sequence alignments of the 46 CpWRKY and 66 CpNAC proteins, as well as the 71 AtWRKY and 132 AtNAC proteins of *A. thaliana*, were performed using the BioEdit program [30] with ClustalX 1.81 (Blosum Weight Matrix; Gap Opening Penalty: 5; Gap Extension Penalty: 0.20) [31]. Likewise, the pHMM from the PFAM database was used to identify the WRKY (https://pfam.xfam.org/family/PF03106 Accessed on 25 July 2022) and NAC (https://pfam.xfam.org/family/PF01849 Accessed on 25 July 2022) domains and were confirmed using the Plant Transcription Factor database V5.0 (PlantTFDB 5.0). The phylogenetic trees were constructed based on the neighbor-joining (NJ) method using the MEGA 11 program (http://www.megasoftware.net/ Accessed on 18 July 2022) [32]. The bootstrap values were calculated using 10,000 bootstrapping replicates and the calculated evolutionary distances were obtained from the Poisson correction evolutionary model. The gaps and missing data were treated with pairwise deletion. The conserved motif structures of the 46 CpWRKY and 66 CpNAC proteins of *C. papaya* were analyzed using the Multiple Expectation Maximization for Motif Elicitation (MEME V5.1.1) program (http://meme-suite.org/tools/meme Accessed on 25 July 2022) with the parameters set by default values.

### 4.3. Cis-Acting Regulatory Elements Analysis and Gene Ontology Annotation (GO)

The identification of the cis-regulatory elements in the putative 5′-UTR and promoter regions of the WRKY and NAC genes families of *C. papaya* and *A. thaliana* (46 CpWRKY and 71 AtWRKY, 66 CpNAC and 132 AtNAC) were conducted on the upstream 2000 bp genomic DNA sequences using PlantCARE (http://bioinformatics.psb.ugent.be/webtools/plantcare/html/ Accessed on 29 July 2022) [33].

### 4.4. Chromosomal Localization 

The chromosomal localization of the 46 *CpWRKYs* and the 66 *CpNACs* within the nine chromosomes of the *C. papaya* genome was obtained from the protein data set from the resequencing of the *C. papaya* genome obtained [26]; The Genome Warehouse (GWH) BioProject: PRJCA006722 AccessionNo. GWHBFSC00000000 https://ngdc.cncb.ac.cn/gwh/Assembly/23161/show Accessed on 14 November 2022. The results were visualized using TBtools [22].

### 4.5. Expression Profiles under Water Deficit Using RNA-Seq Data

The Illumina RNA-Seq data of the commercial-susceptible (cv. Maradol) and wild-tolerant genotypes subjected to water deficit stress (WDS) or well-watered treatment for 3, 7 and 14 days were downloaded from a previous study performed by our working group [16]. In this way, the transcripts that showed the homology with the WRKY and NAC genes were selected.

### 4.6. Experimental Conditions, RNA Isolation and RT-qPCR Analysis

Two *Carica papaya* L. genotypes were evaluated: papaya cv. Maradol (commercial genotype, C) and wild papaya (wild genotype, W), collected in non-perturbated sites in Yucatan, Mexico. Seeds from both genotypes were germinated and seedlings were grown with a mixture of peat moss:soil (1:1) under greenhouse (Mérida, Yucatán) conditions (30–35 °C, 70% RH and photosynthetically active photon flux density (PPFD) of 600 μmol m^−2^ s^−1^). From their germination, they were watered with distilled water every two days for 30 days and 1 mL L^−1^ of Bayfolan (Forte Liquid, Bayer, Germany) was applied twice a week. After 60 more days, the potted plants of the wild and commercial papaya genotypes were separated into four groups—well-watered conditions Group 1: commercial genotype (CW) and Group 2: wild genotype (WW). The other plants were exposed to 0, 3, 7 and 14 days under water deficit conditions, Group 3: commercial genotype (CD) and Group 4: wild genotype (WD) [16]. Each group consisted of five plants in each of the three replicates (*n* = 15) in a completely randomized block design. The fresh leaves that fully expanded were used for the molecular analyses.

The total RNA was extracted from the same tissue using the CTAB protocol and treated with RQ1 RNAse-free DNAse (Promega, Madison, WI, USA) to remove genomic DNA contamination. The concentration and purity of the RNA samples were examined using a NanoDrop^TM^ 1000 Spectrophotometer (Thermo Scientific NanoDrop Technologies, LLC, Wilmington, DE, USA) and the quality was evaluated using 1.5% agarose gel electrophoresis for 30 min at 80 V. For the first-strand cDNA synthesis, Superscript III reverse transcriptase was used, following the manufacturer’s protocol (Invitrogen/Life Technologies, Carlsbad, CA, USA). A set of primers were designed using Primer Express Ver. 3.1 (Applied Biosystems, Waltham, MA, USA) to evaluate the relative expression levels.

Based on their high TPM (transcript per million) and high FC (fold change), c17357_g1_i2 contig (*CpWRKY50*) and c24914_g1_i3 contig (*CpNAC83.1*) were selected for further RT-qPCR analysis. The elongation factor 1−α (*CpEF1α*) was used as a reference gene to normalize all the data. The specificity of each gene was confirmed by the standard melt curve method. The RT-qPCR reactions were performed as reported by [34]. The relative expression levels (REL) of the *CpWRKY50* and *CpNAC83.1* genes were calculated based on the comparative Ct (ΔΔCt) method [35]. The RELs were subjected to a one-way analysis of variance (ANOVA) (*p* < 0.05), compared using Tukey’s test and the Statgraphics Plus 5.1 Software (Statistical graphics Corp., USA).

## 5. Conclusions

In conclusion, the genome of *Carica papaya* has 46 CpWRKY members and 66 CpNAC members, data that confirm that papaya shows a lower number of genes in most TF families than *A. thaliana*. They all showed the characteristic domains and showed various cis-elements related to abiotic stress. In terms of expression analysis in response to water deficit stress, the wild genotype showed a higher expression in response to WDS than the commercial cultivar in a large number of the CpWRKYs and CpNACs analyzed. Our results may suggest that during the domestication process, the ability to respond to drought shown in native (wild) genotypes (including its capacity to overexpress some key *CpWRKY* and *CpNAC* genes in response to drought) was somehow reduced in the current commercial genotypes. This is in agreement with previous suggestions that during the domestication process, many species lost their ability to respond to adverse climatic factors such as drought, high temperatures, salinity, floods, etc. [36,37]. 

## Data Availability

Not applicable.

## Supplementary Materials

The following supporting information can be downloaded at: https://www.mdpi.com/article/10.3390/plants12152775/s1. Table S1: The BLASTP results of the amino acid sequences CpWRKYs found in the *C. papaya* cv. SunUp genome that show the homology to AtWRKYs of *A. thaliana*. The pairwise identity percentage between the 71 AtWRKYs and 46 CpWRKYs proteins. Table S2: The BLASTP results of the amino acid sequences CpNACs found in the *C. papaya* cv. SunUp genome that show the homology to AtNACs of *A. thaliana*. The pairwise identity percentage between the 132 AtNACs and 66 CpNACs proteins. Table S3: The pairwise identity percentage between the amino acid sequences of the 46 CpWRKYs found in the *C. papaya* cv. SunUp genome vs the 29 transcripts of the CpWRKYs type found in the database through the C. *papaya* transcriptome reported by Estrella-Maldonado et al., (2021). Table S4: The pairwise identity percentage between the amino acid sequences of the 66 CpNACs found in the *C. papaya* cv. SunUp genome vs. the 25 transcripts of the CpNACs type found in the database through the C. *papaya* transcriptome reported by Estrella-Maldonado et al., (2021). Figure S1: The multiple sequence alignment of the 71 WRKY proteins of *A. thaliana* against the 46 WRKY proteins identified in *C. papaya* using the program ClustalW. The two conserved motifs (WRKYGQK) and (C2H2 zinc-finger) are pointed. The same color shaded backgrounds indicate partial or entirely conserved amino acid residues, respectively. Figure S2: The multiple sequence alignment clustered only the 46 WRKY proteins identified in *C. papaya* using the program ClustalW. The same color shaded backgrounds indicate partial or entirely conserved amino acid residues, respectively. Figure S3: The multiple sequence alignment of the 132 NAC proteins of *A. thaliana* against the 66 NAC proteins identified in *C. papaya* using the program ClustalW. The same color shaded backgrounds indicate partial or entirely conserved amino acid residues, respectively.
