# Supplementary figures and images for: Genome-Wide Analysis of WRKY and NAC Transcription Factors in Carica papaya L. and Their Possible Role in the Loss of Drought Tolerance by Recent Cultivars through the Domestication of Their Wild Ancestors
Source: Plants (Basel). 2023 Jul 26;12(15):2775. doi: 10.3390/plants12152775 (PMC10421361; doi:10.3390/plants12152775)

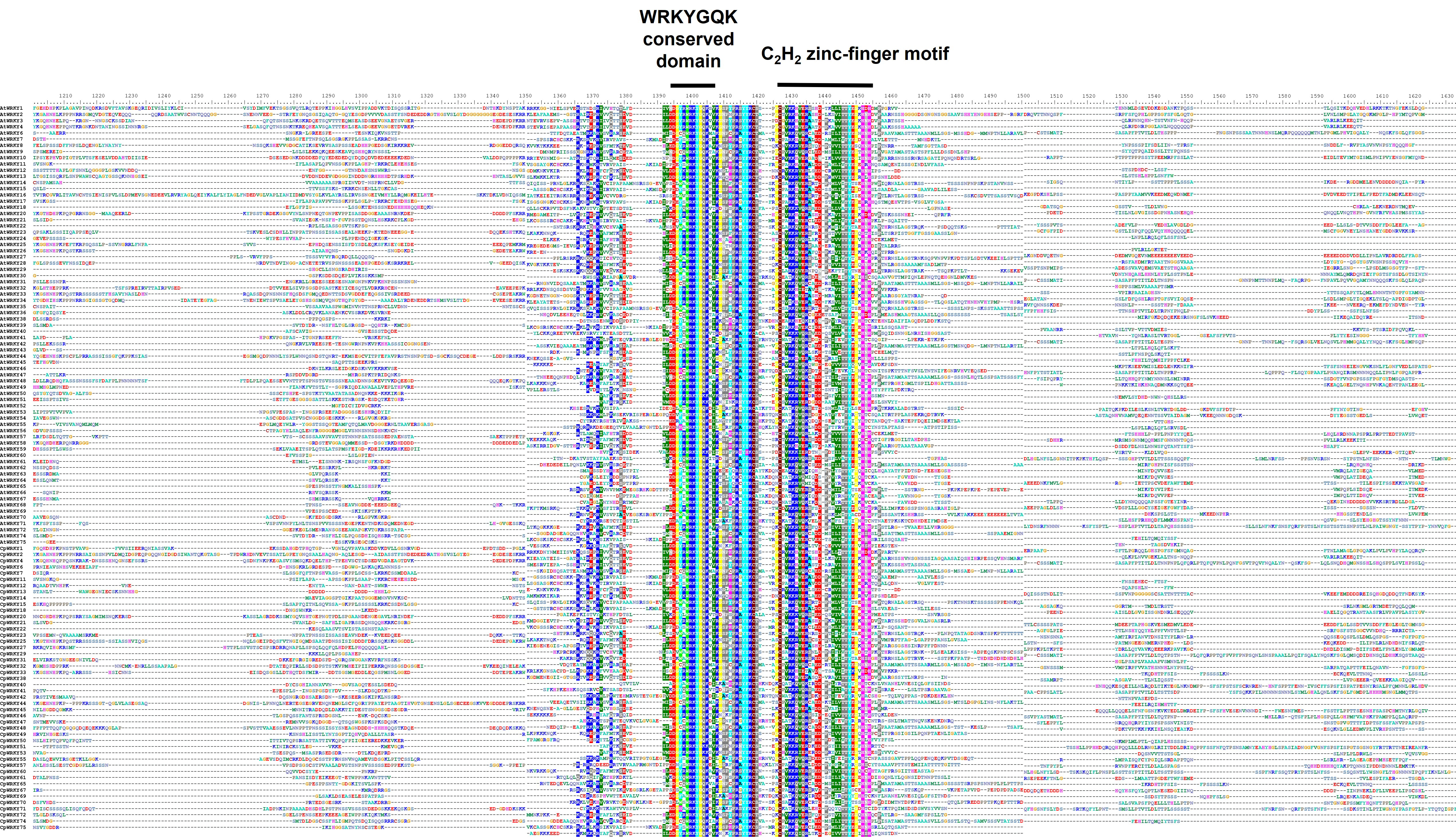

Supplement: Supplementary file 1 [file plants-12-02775-s001.zip › Supplementary Figure S1. The multiple sequence alignment of the 71 WRKY proteins of A. thaliana against the 46 WRKY proteins identified.tif]

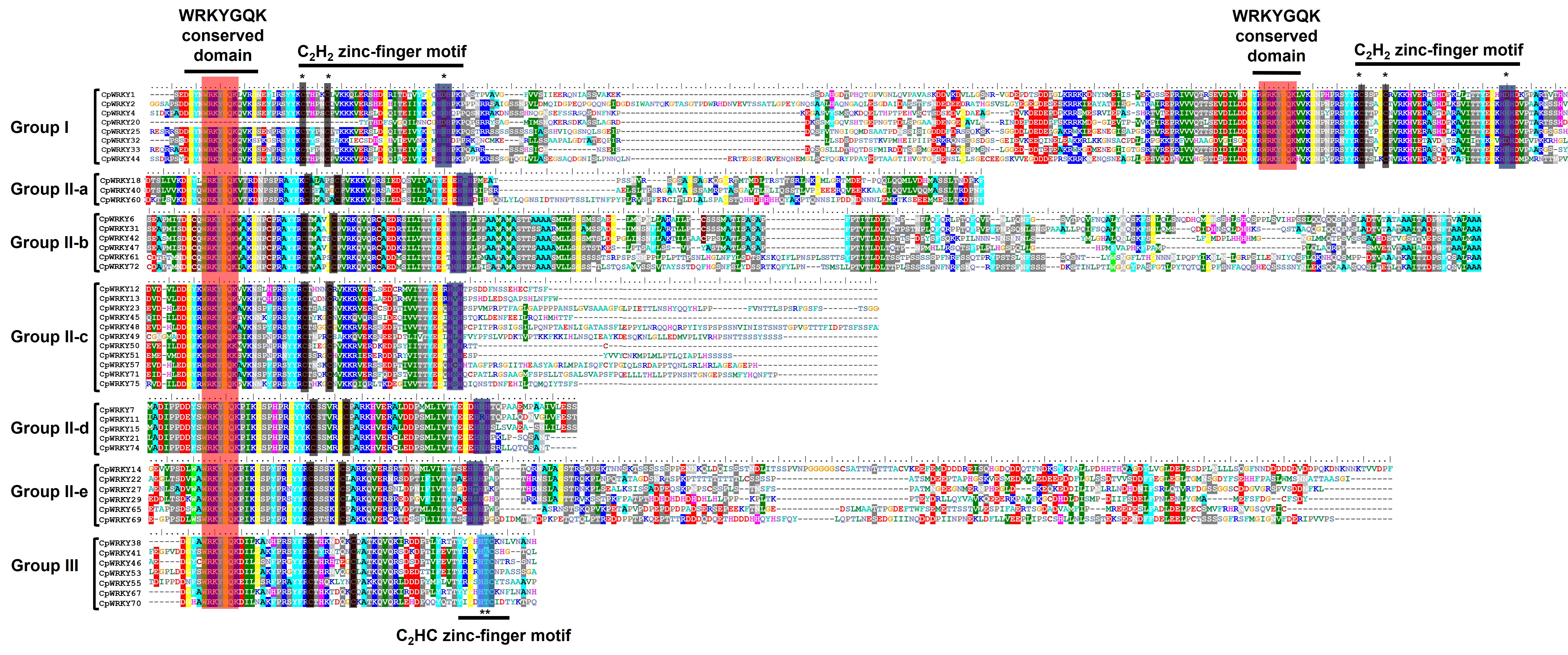

Supplement: Supplementary file 1 [file plants-12-02775-s001.zip › Supplementary Figure S2. The multiple sequence alignment clustered only the 46 WRKY proteins identified in C. papaya using the program .jpg]
